# A Note on the Structural Change Test in Highly Parameterized Psychometric Models

**DOI:** 10.1007/s11336-021-09834-6

**Published:** 2022-02-01

**Authors:** K. B. S. Huth, L. J. Waldorp, J. Luigjes, A. E. Goudriaan, R. J. van Holst, M. Marsman

**Affiliations:** 1 Department of Psychology, University of Amsterdam, Nieuwe Achtergracht 129B, PO Box 15906, 1001 NK Amsterdam, The Netherlands; 2 Department of Psychiatry, Amsterdam University Medical Center, Amsterdam, The Netherlands; 3 Centre for Urban Mental Health, Amsterdam, The Netherlands; 4 Arkin Mental Health Institute, Amsterdam, The Netherlands

**Keywords:** parameter invariance, parameter stability, structural change test, finite sample behavior, permutation test

## Abstract

Equal parameter estimates across subgroups is a substantial requirement of statistical tests. Ignoring subgroup differences poses a threat to study replicability, model specification, and theory development. Structural change tests are a powerful statistical technique to assess parameter invariance. A core element of those tests is the empirical fluctuation process. In the case of parameter invariance, the fluctuation process asymptotically follows a Brownian bridge. This asymptotic assumption further provides the basis for inference. However, the empirical fluctuation process does not follow a Brownian bridge in small samples, and this situation is amplified in large psychometric models. Therefore, common methods of obtaining the sampling distribution are invalid and the structural change test becomes conservative. We discuss an alternative solution to obtaining the sampling distribution—permutation approaches. Permutation approaches estimate the sampling distribution through resampling of the dataset, avoiding distributional assumptions. Hereby, the tests power are improved. We conclude that the permutation alternative is superior to standard asymptotic approximations of the sampling distribution.

**Supplementary Information:**

The online version contains supplementary material available at 10.1007/s11336-021-09834-6.

The assumption of parameter invariance across subgroups—assessed as measurement invariance, differential item functioning, heterogeneity, or parameter stability in other contexts—underlies virtually all statistical tests (Bechger and Maris 2015; Hjort and Koning 2002; Hansen 1997; Mellenbergh 1989). Formally, we can define parameter invariance as,

$$\begin{aligned} f(y\mid v, \theta ) = f(y \mid \theta ), \end{aligned}$$

where $f(\cdot )$ is a parametric distribution that is indexed by a parameter $\theta$, used to model an observed variable *y*, and *v* is an auxiliary variable against which we are testing parameter invariance. Thus, parameter invariance implies that an identical model holds for different subgroups (e.g., males and females, older and younger persons, and persons with different ethnic backgrounds) or across measurement occasions (Putnick and Bornstein 2016; van de Schoot et al. 2015). Violations of this assumption can lead to misspecified models, spurious parameter estimates and test results, therefore, concealing differences key for theory development, diagnostic procedures, and treatment design (e.g., Kapur et al. 2012; Breslau et al. 2008). Unfortunately, researchers often neglect parameter invariance, which poses a threat to research development (Borsboom 2006).

Structural change tests (SCTs) allow us to assess parameter invariance across subgroups (Brown et al. 1975). These tests were initially proposed by Andrews (1993) for parameter stability assessment in econometric time-series models, but since then have been adapted to assess models across the statistical sciences (e.g., Chang and Su 2014; Mulaudzi 2016; O’Connell et al. 2018; Strobl et al. 2015; Zeileis et al. 2008; Merkle et al. 2014). SCTs have become a popular method for assessing parameter invariance because they can be straightforwardly implemented, even for complicated psychometric models: SCTs do not require explicit specification of which parameter diverges or which subgroups behave differently (Wang et al. 2018). Different type of SCTs exist that differ in the particular type of influence measure used [e.g., recursive residuals (Brown et al. 1975) or single-shift test statistics (Andrews 1993)], but depicting closely related asymptotic properties. In this paper, we focus on SCTs using *scores* as influence measures (Merkle and Zeileis 2013; Zeileis 2006; Hjort and Koning 2002). Scores are partial derivatives of the log-likelihood function with respect to a particular parameter and can be considered similar to asymptotic influence functions which are used to determine the effect of single observations on the estimate (Hampel et al. 2005). SCTs assume that if parameter invariance holds, aggregated scores randomly fluctuate about zero and converge to a Brownian bridge (Hjort and Koning 2002); a process that starts and ends at zero and randomly fluctuates about zero in between with its individual elements being normally distributed. However, if the aggregated scores systematically change in line with changes of an auxiliary variable *v*, parameter invariance is violated (Zeileis 2006). This result is used to determine the sampling distribution of the SCT’s test statistic.

The test statistic’s sampling distribution is well-determined for large sample sizes (Hansen 1997; Estrella 2003). However, in finite samples, concerns arise. For a simple linear model, the test shows both sub-optimal power and a type 1 error rate below the expected nominal $\alpha$-level (Zeileis and Hothorn 2013). The asymptotic sampling distribution does not exploit the significance level and is of poor quality compared to the exact conditional and conditional asymptotic distribution (Hothorn and Zeileis 2008; Zeileis and Hothorn 2013). In short, the SCT becomes increasingly conservative in finite samples (Jones et al. 2020; Merkle and Zeileis 2013; Strobl et al. 2015). In this paper, we delve into this issue, focusing on large psychometric models. Our goals are twofold. Our first goal is to assess the SCT’s behavior in finite samples. In particular, we investigate the sampling distribution and the distribution of the *p* value, which should be uniformly distributed under the null hypothesis. We show that for finite samples, the sampling distribution is misspecified and *p* values are not uniformly distributed. This problem becomes more pronounced the larger the model (i.e., the more parameters to be estimated). Our second goal is to show a solution to this misspecification—permutation approaches—allowing for the SCTs application in large psychometric models. Permutation approaches are useful for estimating the sampling distribution when distributional assumptions do not hold or are analytically intractable (Mooney and Duval 1993). We show that permutation approaches provide a correct type I error and tend to increase the test’s statistical power in finite samples (Zeileis and Hothorn 2013). In this way, permutation methods are superior to standard asymptotic approaches.

The remainder of this paper is organized as follows. First, we introduce the SCT in detail. Second, we investigate the SCT’s finite sample behavior, and in particular, the distribution of *p* values under the null hypothesis. Here, we establish that the asymptotically derived sampling distribution is incorrect for finite sample sizes. Third, we elaborate on an alternative approach to obtaining the sampling distribution—permutation approaches. To illustrate the issues and our solution, we will use a linear regression model and a Gaussian Graphical model throughout this paper.

## Structural Change Tests

The SCT assesses the equivalence of all *k* model parameters (i.e., $j = 1, \ldots , k$) across subgroups defined by an auxiliary variable *v* (Andrews 1993). Under the null hypothesis, the SCT assumes that a parameter $\theta _j$ is the same for all subgroups $v_g$, $g = 1,\dots ,m$, of the auxiliary variable. That is,

$$\begin{aligned} \mathcal {H}_0:\, \,\theta _{j\,{v_g}} = \theta _j; \forall \, 1\le g\le m, 1\le j \le k, \end{aligned}$$

where $\theta _{j\,{v_g}}$ denotes the parameter value of subgroup $v_g$ for parameter $\theta _j$. The SCT comprises three steps: First, one estimates the model of interest and determines its parameter influence measure. Here, we focus on score-based SCTs (Hjort and Koning 2002; Zeileis and Hornik 2007). Secondly, so-called empirical fluctuation processes are derived from the scores. Thirdly, the fluctuation processes are aggregated into a test statistic and compared against the sampling distribution to compute the *p* value. We outline each of these steps below.

The first step consists of estimating the *k* parameters of a model of interest. This paper will focus on estimating the model parameters through maximum likelihood estimation (MLE; for other approaches, see, for example, Kuan and Hornik 1995). With the MLEs, the score for every particular parameter and observation can be calculated. The score is the gradient of the log-likelihood function and for a parameter $\theta _j$ and observation $y_i$, it is denoted by

$$\begin{aligned} \text {s}(\theta _j, y_i) = \frac{\partial \log \text {L} (\theta ; y_i) }{ \partial \theta _j} , \end{aligned}$$

where $\text {L}$ is the likelihood function of the model, $\theta _j$ the focal parameter and $y_i$ the data for an observation *i*. Since the MLEs maximize the log-likelihood function, the sum of the scores for a parameter *j* across all *n* observations will sum to zero:

1 $$\begin{aligned} \sum _{i = 1}^{n} \text {s} (\hat{\theta }_j, y_i) = 0, \end{aligned}$$

which holds for all parameters in the model.

In the second step, the accumulations of scores across observations are interpreted as empirical fluctuation processes. These fluctuation processes are analyzed separately for every parameter of the model. To obtain the fluctuations, the scores are first ordered along the auxiliary variable *v* and then aggregated across observations:

$$\begin{aligned} \Psi (t; \hat{\theta }_j) = n^{-1/2} \sum _{i = 1}^{ \lfloor n t \rfloor } \text {s}(\hat{\theta }_j,y_i), \end{aligned}$$

where $\lfloor nt \rfloor$ is the floor function of $n \times t$. Note, *t* is a real number and in practice with discrete measurements commonly a proportion, such as here a fraction of the *n* participants (i.e., $t = i/n$ for ). $\sum _{i = 1}^{ \lfloor nt \rfloor }$ therefore describes the sum of all scores up until the $(n \times t)-$th term, which is referred to as the cumulated score. To ensure that the cumulative scores are independent across parameters, $\Psi (t\text {; } \hat{\theta }_j)$ is decorrelated (Merkle and Zeileis 2013):

$$\begin{aligned} \text {B}(t; \hat{\theta }) = \hat{I}^{-1/2} \Psi (t; \hat{\theta }), \end{aligned}$$

where ${\hat{I}}$ is the asymptotic covariance matrix of the scores, thus, an estimate of the Fisher information matrix (Zeileis 2006). Observe that the cumulated scores $\text {B}(t; \hat{\theta }_j)$ are zero for $t = 0$ and also at $t = 1$. At $t = 1$, the scores of all observations have been summed up, which by definition of the MLE is zero, e.g., Eq. (1).

**Fig. 1:**
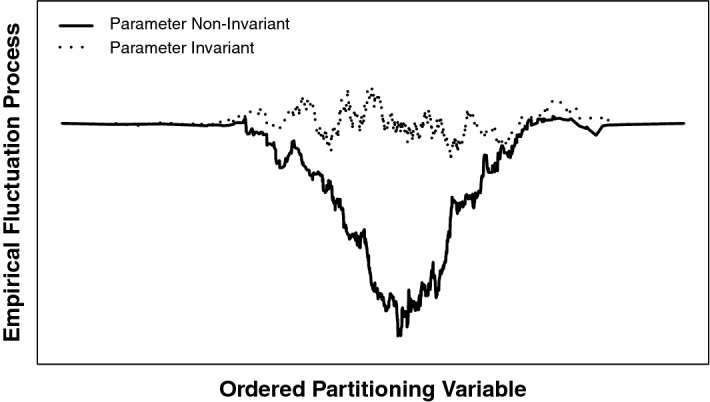
Visualization of empirical fluctuation processes for two exemplary parameters. The dotted line represents the cumulative scores for a parameter with a random fluctuation around zero; thus, the fit for that parameter does not depend on the auxiliary variable. The solid line represents a systematic fluctuation coinciding with the auxiliary variable; parameter invariance is violated.

Under $\mathcal {H}_0$, the fluctuation processes asymptotically converge to a Brownian bridge (Hjort and Koning 2002; Andrews 1993) and for a model with *k*-parameters to *k*-independent Brownian bridges,

$$\begin{aligned} \text {B}(\cdot \text {; } \hat{\theta }) \xrightarrow {d} \text {B}^0(\cdot ), \end{aligned}$$

where $\xrightarrow {d}$ denotes weak convergence of $\text {B}(\cdot ; \hat{\theta })$ to a *k*-dimensional Brownian bridge $\text {B}^0(\cdot )$. Parameter stability can now be visually assessed by plotting the fluctuation process. The fluctuation process randomly varies about zero if $\mathcal {H}_0$ were true and parameter invariance holds. However, in the case of parameter non-invariance, the process systematically deviates from zero. Figure 1 provides an illustration.

In the third and final step, the cumulative scores are combined into a test statistic which can be conducted in various ways (Merkle and Zeileis 2013; Hjort and Koning 2002). We will introduce the three test statistics commonly used in literature: The double maximum statistic (DM), the Cramér–von Mises statistic (CvM), and the maximum Lagrange Multiplier statistic (maxLM).234where $\text {B}_{ij}$ denotes the fluctuation process at an observation *i* for a parameter $\hat{\theta }_j$, i.e., $\text {B}_{ij} = \text {B}(t = \frac{i}{n}; \hat{\theta }_j)$. The DM statistic takes the maximum of the cumulated scores across observations and parameters, and is used to test if any fluctuation process deviates too strongly from zero at any time. The CvM captures fluctuations that change across a variety of observations and parameters. Lastly, the maxLM statistic is suited if all *k* fluctuation processes change along the same observation *i*. To circumvent precision issues, the fluctuation process’s tails are not considered when computing the maxLM statistic.

In null hypothesis significance testing, the test statistic computed from observed data is compared against the sampling distribution to obtain a *p* value. Critical values can be obtained by simulating observations from a Brownian bridge and applying the relevant statistic to the generated data (Andrews 1993; Zeileis 2006). Also, closed form solutions exist for specific situations. For example, Ploberger and Kramer (1992) derive the sampling distribution for DM-type statistics and Hjort and Koning (2002) show that CvM-type statistics follow an approximate $\chi ^2$-distribution that depends on the amount of parameters and focal change point assessed. Furthermore, Hansen (1997) established for the maxLM statistic that if the focal parameter changes close to the half-point of the auxiliary variable, the sampling distribution converges to a $\chi ^2$-distribution. Hansen presented critical statistics and *p* values for specific combinations of model parameters *k* and change point location. Estrella (2003) extends those results for high-dimensional scores. We will use the simulation method for determining the sampling distribution (Zeileis 2006).

## Small Sample Behavior of the Structural Change Test

Simulations of the SCT’s behavior concluded that the SCT shows suboptimal behavior in finite samples (Hothorn and Zeileis 2008; Merkle and Zeileis 2013; Strobl et al. 2015; Jones et al. 2020). Its power dwindles in finite samples and surprisingly, the type 1 error decreases with increasing model complexity, staying below the respective significance threshold. To provide a better understanding of the nature and severity of this issue as well as potential ways to mitigate it, we will delve into the problem. We will analyze it in two ways, first through simulation and second through mathematical derivation of the convergence rate.

### Simulation of Small Sample Behavior

We will analyze the SCT’s behavior through simulation for two models: A simple linear regression model and a more complex Gaussian graphical model (GGM). Our simulations vary the sample size *n* and the covariates/nodes in the models. For the linear regression, we simulated models with two, four, and eight covariates (i.e., regression coefficients) for 50, 200, and 1000 observations each. For the GGM, we simulated networks with five, ten, and fifteen nodes for 200, 500, and 2000 observations. Each combination was run 5000 times. Datasets were simulated as a multivariate normal distribution $\mathcal {N}( \mu , \Sigma )$ with a sparse interaction matrix $\Sigma$ (i.e., probability of interaction was 0.2) without any dependency on an auxiliary variable. Thus, data were generated under the null hypothesis of equal parameters across subgroups. All simulations were run in the software R (R-Core-Team 2020); the SCT was conducted using the strucchange function for the linear regression model (Zeileis et al. 2002) and the partykit::mob function for the GGM (Zeileis et al. 2008; Hothorn and Zeileis 2015).

**Fig. 2:**
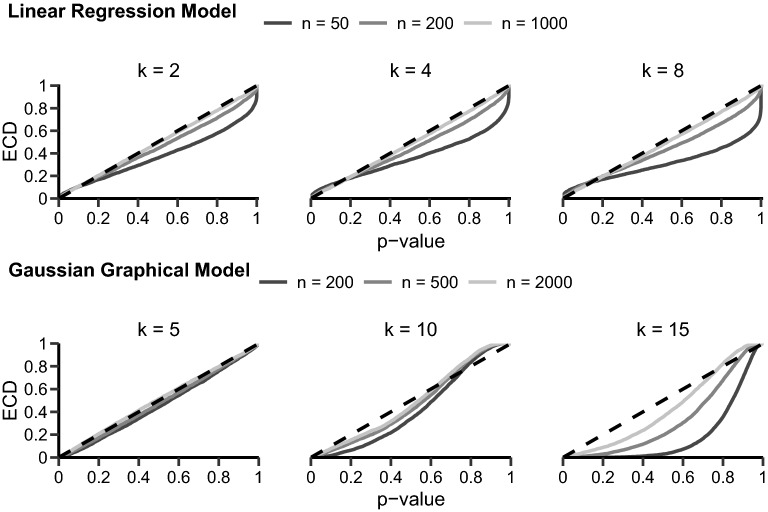
Empirical cumulative distributions (ECDs) for the *p* value under the null hypothesis for different models and simulation settings. The top row shows the linear regression model results and the bottom row shows the GGM results. Here, *n* denotes the sample size and *k* the number of covariates for the linear regression model and the number of nodes for the GGM. In each plot, the black, dashed line shows the expected uniform distribution.

We will focus on the results for the maxLM statistic with a continuous auxiliary variable. In the online appendix, we report the results for a binary auxiliary variable as well as the results for CvM and DM statistics.^1^ The simulated *p* value distributions are shown in Fig. 2. The *p* value is expected to follow a uniform distribution under the null hypothesis, which is indicated with the dashed, black line in each of the plots in Fig. 2. Observe that the *p* values do not follow this uniform distribution for the linear regression model in the smaller sample sizes but approximate a uniform distribution if the sample size increases. For the GGM, the *p* value is nearly uniformly distributed in small networks for all sample sizes. However, for larger networks, the *p* value distribution deviates. A result that appears to be independent of the sample size used in our simulations. The deviation between the simulated *p* value distribution and the correct uniform distribution is largest for networks with 15 nodes and 200 observations; here, even with 2000 observations, the *p* value does not follow a uniform distribution.

**Fig. 3:**
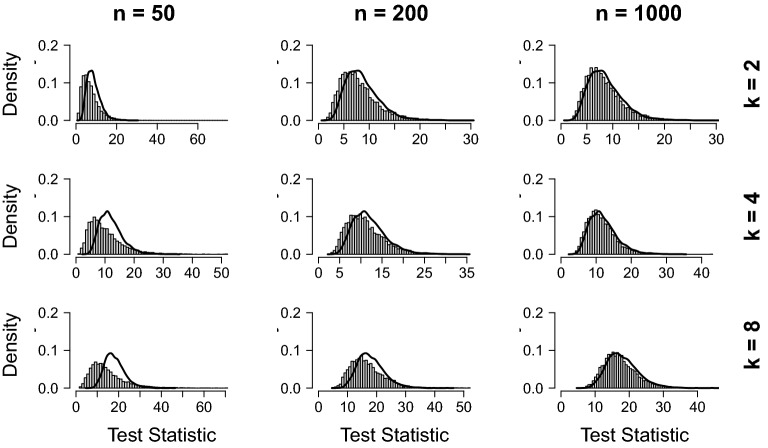
Distributions of the maxLM statistic under the null hypothesis for the linear regression model. The expected sampling distribution is depicted as a black line and was obtained by simulating observations from a Brownian bridge and applying the maxLM statistic to them (e.g., see Zeileis 2006).

**Fig. 4:**
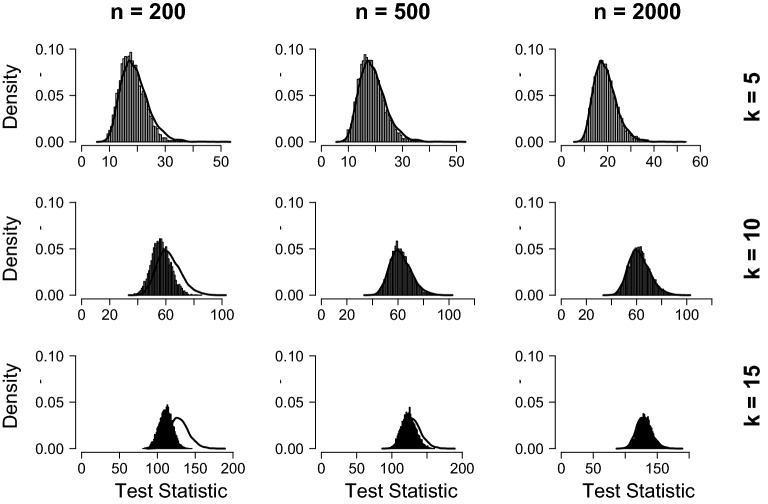
Distributions of the maxLM statistic under the null hypothesis for the GGM. The expected sampling distribution is depicted as a black line and was obtained by simulating observations from a Brownian bridge and applying the maxLM statistic to them (e.g., see Zeileis 2006).

The simulated sampling distributions are shown in Fig. 3 for the linear regression model and in Fig. 4 for the GGM. The asymptotic sampling distributions are indicated with a black solid line in these graphs. They were generated by repeatedly simulating values from a Brownian bridge and then computing the maxLM statistic on the generated data (e.g., see Andrews 1993; Zeileis 2006). In computing the maxLM statistic, a choice is made to cut off the empirical fluctuation process’s tails to avoid precision issues. The choice of cut-off points can, in principle, improve the fit of the estimated sampling distribution and lead to cherry-picking cut-offs that improve the fit of the sampling distribution. For the GGM, we chose to cut off the process’s tails before $n_p$ and after $n - n_p$ observations, respectively, where $n_p$ denotes the number of free parameters in the model (i.e., similar to Jones et al. 2020). For the linear model, we chose to cut-off the bottom and upper 10%. Results in Figs. 3 and 4 show clearly that for the linear regression model, the sampling distribution is specified correctly for larger sample sizes, independent of model complexity, but not for smaller sample sizes. For the GGM, the sampling distribution is properly specified for small networks, but large discrepancies are found for larger networks.

From the assessment thus far, the true nature of the finite sample misspecification is unknown. The k-independent Brownian bridges approximation and thereby the correct specification of sampling distribution depends on two assumptions: the normal approximation to the score distribution and the accuracy of the estimated information matrix. In order to assess these two fundamental assumptions, we conducted some small scale simulations. Results indicate that the scores of a threshold parameter do follow a normal distribution fairly well for all number of nodes and observations (see Figure S5 in the online appendix). However, for an interaction parameter the score distribution deviates highly from the expected one; the distributions are skewed, multi-modal, and peaked (see Figure S6 in the online appendix). Furthermore, the estimated Fisher information matrix is biased. However, the bias reduces with increased sample size, especially for larger models (see Figure S7 in the online appendix). A detailed description of the results can be found in the online appendix.

In sum, the *p* value and test statistic do not follow the expected distributions in finite samples for the simple linear regression model and the more complex GGM. This problem was especially pronounced for small sample sizes in combination with complex models. Both fundamental requirements—the normal distribution of the scores and the unbiased estimation of the Fisher information matrix—are not met, however, even in setups where the sampling distribution seems properly specified.

### Formal Analysis of the Approximation Error and Convergence Rate

Now, we turn to a formal analysis of this error and derive the convergence rate of the normal approximation for the fluctuation process. The full analysis can be found in Appendix.

Hjort and Koning (2002) used a linear (i.e., second-order) Taylor expansion to derive the normal approximation of the fluctuation process for $\hat{\theta }$ near $\theta _0$, which tends to be accurate in large samples. They furthermore show that the aggregated fluctuation process (i.e., the canonical monitoring process) approximates several independent Brownian bridges under the null hypothesis (see Hjort and Koning 2002 Eqs. (2.3) and (2.4), p. 116]). This approximation provides the basis to derive the SCT’s sampling distribution. Hence, the sampling distribution will be valid (i.e., have correct type I error rate), if the error of the normal approximation of the fluctuation process goes to zero sufficiently fast. The error of the approximation can be assessed by looking at the Lagrange remainder. Whereas Hjort and Koning ignored this Lagrange remainder in their derivations, we will assess the rate at which it convergences to zero.

The Lagrange remainder of a linear (i.e., second-order) Taylor approximation is defined as

$$\begin{aligned} E_2(\theta ) = \frac{f^{''}(\theta _s)}{2} (\hat{\theta } - \theta _0)^2, \end{aligned}$$

for $\theta _s$ between $\theta _0$ and $\hat{\theta }$. Therefore, the full Taylor expansion for the fluctuation process is:

$$\begin{aligned} \Psi (t\text {; } \hat{\theta }) = \overbrace{\frac{1}{\sqrt{n}} \sum _{i = 1}^{ \lfloor nt \rfloor } s( y_i, \theta _0) + \frac{1}{\sqrt{n}} \sum _{i = 1}^{ \lfloor nt \rfloor } i( y_i, \theta _0) (\hat{\theta } - \theta _0)}^{\text {Linear Taylor Approximation}} + \overbrace{\frac{ 1}{2 \sqrt{n}} \sum _{i = 1}^{ \lfloor nt \rfloor } j( y_i, \theta _s) (\hat{\theta } - \theta _0)^2}^{\text {Lagrange Remainder}} , \end{aligned}$$

where $j( y_i, \theta )$ denotes the third-order derivative of the log-likelihood function (i.e., the second-order derivative of the score function). The Lagrange remainder consists of two parts $(\hat{\theta } - \theta _0)^2$ and $j( y_i, \theta _s)$. Observe that $(\hat{\theta }-\theta _0)^2$ is the standard error of the estimator, which tends to zero at rate $\frac{1}{\sqrt{n}}$ for an unbiased or asymptotically unbiased estimator. In Appendix, we show that the third-order derivative $j( y_i, \theta _s)$ for exponential family models, such as normal linear regression model and the GGM, is constant, if their moments are bounded. As a result, the Lagrange remainder is bounded by $\frac{1}{\sqrt{n}}$, thus tends to zero for sufficiently large sample sizes. Unfortunately, this convergence rate is not very fast, which means that the approximation error could be significant; thus, the fluctuation process cannot be accurately described using the Brownian bridge. In this case, the sampling distribution is misspecified (Zeileis 2006; Estrella 2003; Hansen 1997; Hjort and Koning 2002) and the reported *p* value is wrong.

## A Monte Carlo Permutation Approach to the Structural Change Test

Permutation testing is a popular nonparametric method for statistical testing if distributional assumptions are not met. Zeileis and Hothorn (2013) used a permutation test approach to increase the power of the SCT in small samples for linear regression models. Even though their results were positive, the permutation test alternative to the asymptotic version of the SCT has found limited application. Here, we want to assess if a permutation test approach can estimate the correct sampling distribution in finite samples, even for large psychometric models, and consequently control the type I error rate. In permutation tests, first introduced by Fisher (1951), sampling distributions are obtained by calculating the test statistic values under all possible rearrangements of the observed data points. Applied to the SCT, it would thus consider all *n*! rearrangements of the auxiliary variable *v*, and then compute a test statistic for every possible arrangement. Since the labels are exchangeable under the SCT’s null hypothesis, the permutation test approach provides exact significance levels (Good 1993). Compared to parametric tests (e.g., the *t*-test, or *F*-test), permutation tests are equally powerful in large samples (Bickel and van Zwet 2012); however, permutation approaches are more powerful, if assumptions of the parametric tests are not met. The permutation approach’s major drawback is that recomputing the statistic for all possible rearrangements can become unwieldy. A Monte Carlo approach has been proposed in which possible rearrangements are randomly sampled (Kaiser 2007; Frank and Witten 1998). This alternative overcomes the exact permutation tests’ computational burden and provides an approximate permutation test. We will use the approximate permutation test approach and illustrate that it provides accurate sampling distributions, even for small sample sizes and large psychometric models.

The Monte Carlo permutation approach to the SCT comprises three steps. The test statistic for the original dataset is computed in the first step. We will consider the maxLM test statistic in Eq. (4) here. In the second step, we randomly rearrange the values of the grouping variable *v*. For example, say we have an original dataset with six observations belonging to two subgroups (i.e., Group A: 1, 2, and 3; Group B: 4, 5, and 6). After rearranging, observations three, four, and six might now belong to Group A and observations one, two, and five to Group B (i.e., Group A: 3, 4, and 6; Group B: 1, 2, 5). The maxLM test statistic is computed for every random rearrangement. We have used 5,000 random rearrangements in our simulations. It gave a good trade-off between accuracy and computation speed; however, the more samples are obtained, the more accurate the determined *p* value. In the final step, we estimate the *p* value by calculating how many resampled test statistics were larger than the original statistic.

**Fig. 5:**
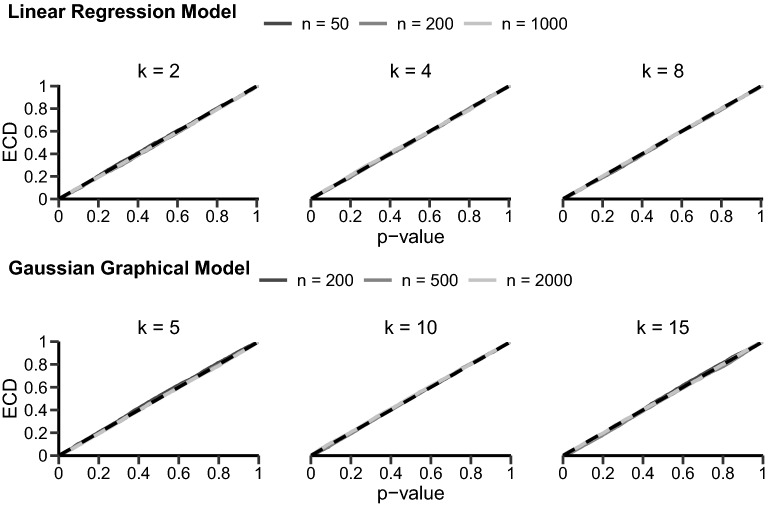
Empirical cumulative distributions (ECDs) for the *p* value under the null hypothesis using the permutation approach. The top row shows the linear regression model results and the bottom row the results for the GGM. Here, *n* represents the sample size and *k* the number of covariates for the linear regression model and number of nodes for the GGM. In each plot, the black, dashed line shows the expected uniform distribution.

We revisit the previous section’s simulations to illustrate the SCT’s behavior when combined with the Monte Carlo permutation test approach. The results are shown in Fig. 5. It is evident that the *p* values now nicely follow a uniform distribution in all simulation setups. No differences can be found depending on sample size or model complexity. Thus, the permutation test approach has solved the underlying issue of the misspecified sampling distribution. Furthermore, we have investigated the power of the SCT under the permutation alternative for GGMs. The effect of the permutation approach on the power of the SCT has been studied before for linear models (e.g., Hothorn and Zeileis 2008; Zeileis and Hothorn 2013). Previous results indicate that permutation testing leads to small power improvements in large samples but leads to huge power improvements in small samples. We show the results of our analyses for GGM in Table 1. In line with previous results for linear models, we find that the power of the permutation test approach is similar to the power based on the asymptotic sampling distribution for large samples. In small samples, however, we see considerable improvements.

**Table 1:** Power of the SCT using the common asymptotic approach and the permutation alternative

		k = 5			k = 10			k = 15		
*n*	Distribution / $\Delta \theta =$	0.1	0.3	0.5	0.1	0.3	0.5	0.1	0.3	0.5
200	Asymptotic Approach	0.57	0.85	0.96	0.01	0.02	0.21	0.00	0.00	0.00
	Permutation Alternative	0.60	0.85	0.97	0.05	0.15	0.52	0.03	0.10	0.19
500	Asymptotic Approach	0.70	0.98	1.00	0.03	0.30	0.94	0.01	0.02	0.31
	Permutation Alternative	0.69	0.98	1.00	0.07	0.45	0.98	0.07	0.22	0.65
2,000	Asymptotic Approach	0.90	1.00	1.00	0.13	0.99	1.00	0.06	0.86	1.00
	Permutation Alternative	0.92	1.00	1.00	0.19	1.00	1.00	0.09	0.91	1.00

We altered sample size (i.e., n = 200, 500, and 2,000), amount of nodes (i.e., k = 5, 10, and 15) as well as the size of the parameter invariance (i.e., $\Delta \theta$ = 0.1, 0.3, and 0.5). Datasets were simulated using a similar simulation setup as Jones et al. (2020).

Based on these results, we conclude that the Monte Carlo permutation approach is a valuable method to perform the SCT, particularly for large psychometric models and small sample sizes. It leads to correct specification of the sampling distribution under the null hypothesis and tends to improve power.

Unfortunately, the permutation alternative comes with high additional computational efforts, which might hamper the use of the permutation approach in large models. However, the biggest misspecification is established for larger models and thus the permutation approach has the greatest benefit here. Therefore, alternatives should be considered that allow for the assessment of parameter invariance but employ sound inference also in finite samples. As a concrete example, we could feed the SCT’s scores into the conditional inference tree (CTree; Hothorn et al. 2006). CTree is a recursive partitioning algorithm assessing parameter invariance using permutation approaches to obtain the sampling distribution (Schlosser et al. 2019). The algorithm evaluates the association between the transformed responses *h*(*Y*) and each of the transformed splitting variables $g(Z_j)$. CTree requires the specification of an influence function $h(\cdot )$ and the transformed split variable function $g(\cdot )$. In case a parametric model is fitted to the observed data, one can obtain a model-based transformation function $h(\cdot )$, for example, a score-function. Here, CTree closely resembles the SCT, however, leveraging a conditional inference framework. Through simulations, we could confirm that CTree circumvents the small sample issues in finite samples also for large models; the *p* value distribution is uniform for all setups of *k* and *n* (see Figure S8 in the online appendix). Thus, CTree serves as an additional solution to circumvent a misspecified *p* value in small samples, which is readily implemented in the R-package networktree for GGMs (Jones et al. 2020).

## Discussion

This paper has shown that the score-based structural change test’s (SCT’s) small sample behavior can be problematic, especially for large psychometric models. The SCT assumes that the accumulation of scores for a parameter across observations resembles a Brownian bridge. This property holds for large samples but not for small samples. As a result, standard methods that rely on this asymptotic property cannot determine the SCT’s correct sampling distribution. However, using a Monte Carlo permutation test approach, the proper sampling distribution can be obtained. As a result, the correct *p* values can be determined even for small samples and large psychometric models. This permutation approach also improves the power of the test.

Previous research has observed finite sample problems for the SCT, concluding that the SCT constitutes a conservative test with decreasing sample size (Strobl et al. 2015; Merkle and Zeileis 2013; Hothorn and Zeileis 2008). The SCT’s sampling distribution is misspecified in small samples, which leads to problems with controlling the type I error, thus, incorrect inference. These findings are concerning. In particular, since the SCT has been adapted to larger psychometric models where small sample issues are amplified (e.g., Strobl et al. 2015; Jones et al. 2020). These adaptations additionally combine large models with the model-based recursive partitioning (MOB). The MOB is a recursive algorithm that uses the SCT to detect parameter invariance and splits data into smaller subsets for which it recalculates the SCT (Merkle and Zeileis 2013). When using subsets of the data in recursive applications of the SCT, its small sample properties can become more pronounced. In sum, the small sample properties of the SCT are a timely matter.

The Monte Carlo permutation approach offers a straightforward alternative to obtain the sampling distribution. We showed that the alternative approach leads to a correct specification of the sampling distribution, and consequently, a correct specification of the *p* value distribution. Also, we were able to corroborate previous demonstrations of the increased power of the permutation approach over asymptotic approximations in linear models (Hothorn and Zeileis 2008; Zeileis and Hothorn 2013), by extending these results to the GGM. We found higher power for the permutation approach, especially for large psychometric models combined with small sample sizes. Therefore, the permutation approach solves the issue of the misspecified sampling distribution and increases the power of the test, leading to an optimal result in finite samples.

The permutation test algorithm comes with some drawbacks. First, the algorithm has a highly increased computational effort. Our current implementation is in base R (R-Core-Team 2020) and could be improved through computationally more efficient programming languages like the Rccp plugin for R (Eddelbuettel and François 2011). Second, researchers should be wary that the permutation results depend on the number of permutations and random seeds of the sampling algorithm. It is therefore advised to use a sufficiently large number of permutations and test different random seeds. Nonetheless, by solving the misspecified sampling distribution and increasing the power, we conclude—similar to previous researchers—that the additional computational power needed for the permutation approach is justified and necessary in finite samples (Hothorn and Zeileis 2008; Zeileis and Hothorn 2013).

Still, researchers should consider alternative parameter invariance tests. In the paper, we discussed the readily implemented algorithm CTree (Schlosser et al. 2019; Hothorn et al. 2006). CTree provides a correct *p* value distribution also in finite samples, however, has even less power than the asymptotic SCT algorithm. Thus, if researchers are mainly concerned with a high power, it would still be advisable to apply the permutation approach to the SCT. Other alternatives could still be derived. Here, we need to understand the nature of the problem; in particular, the two fundamental requirements: the normal distribution of the scores and the unbiased estimation of the information matrix. Our preliminary assessment showed that the scores of the interaction parameters do not follow a normal distribution and the bias of the Fisher information matrix is inflated. Astoundingly, the assumptions are not met, even in situations where the sampling distribution is properly specified. More research is needed to understand the nature of this issue and perhaps offer parametric alternatives for the sampling distributions.

To conclude, finite sample misspecification of the structural change test needs to be acknowledged. Here, permutation approaches are a superior method to standard asymptotic approximations of the sampling distribution. Especially in large psychometric models, a wider adaptation of permutation approaches for SCTs is advisable.

### Supplementary Information

Below is the link to the electronic supplementary material.

Supplementary file 1 (pdf 1241 KB)

## Appendix: Formal Analysis of the Approximation Error and Rate of Convergence

We derive the error associated with the Brownian bridge approximation of the fluctuation process. First, we introduce the derivation of this approximation as shown in Hjort and Koning (2002). Second, we derive the error associated with the approximation, the Lagrange remainder in the Taylor approximation and conclude its convergence rate is bounded by $1/\sqrt{n}$.

### The Cumulative Score Process

Let $s( y_i, \theta )$ denote the first-order derivative of the log-likelihood function *g* with respect to $\theta$—the score—and $i( y_i, \theta )$ the second-order derivative. To determine whether scores fluctuate along a third variable of interest (e.g., gender, time), we compute the cumulative sum of the score:

$$\begin{aligned} \Psi (t; \theta _0) = \frac{1}{\sqrt{n}} \sum _{i = 1}^{ \lfloor nt \rfloor } s( y_i, \theta _0), \end{aligned}$$

where $\lfloor nt \rfloor$ is the floor function of $n \times t$—index *n* the sample size and index *t* a fraction of all *n* participants (i.e., $t = i/n$ for ). Here, $\theta _0$ describes the parameter estimate under the null-hypothesis. The mean of the cumulative score process is zero and the variance the information matrix $J = - E(i( y_i, \theta _0))$. Given the Donsker and Cramér-Wold Theorem, one can derive

$$\begin{aligned} \Psi (t; \theta _0) \xrightarrow {d} Z_0(t) \ \ \text {in} \ \ D_p[0,1], \end{aligned}$$

where $Z_0(t)$ is a zero-mean Gaussian, which is a linear transformation of independent Brownian motions (Hjort and Koning 2002). This convergence takes place in the the space $D_p[0,1]$ thus for *t* being in the range zero to one (i.e., $t \in [0, 1]$).

### The Estimated Cumulative Score Process

Given that $\theta _0$ is commonly unknown, we use the maximum likelihood estimator (MLE)—$\hat{\theta }$—and calculate the cumulative score process as

$$\begin{aligned} \Psi (t\text {; } \hat{\theta }) = \frac{1}{\sqrt{n}} \sum _{i = 1}^{ \lfloor nt \rfloor } s( y_i, \hat{\theta }). \end{aligned}$$

For MLE estimators the cumulative score process is bounded at zero, both at $t = 0$ and $t = 1$. Using a Taylor expansion up to the first-order derivative, e.g., which for a function *f* would be,

$$\begin{aligned} f(\hat{\theta }) = f(\theta _0) + f'(\theta _0) (\hat{\theta } - \theta _0), \end{aligned}$$

Hjort and Koning approximate the cumulative score process for $\hat{\theta }$ near $\theta _0$ as

$$\begin{aligned} \Psi (t; \hat{\theta }) \doteq \frac{1}{\sqrt{n}} \sum _{i = 1}^{ \lfloor nt \rfloor } s( y_i, \theta _0) + \frac{1}{\sqrt{n}} \sum _{i = 1}^{ \lfloor nt \rfloor } i( y_i, \theta _0) (\hat{\theta } - \theta _0) \end{aligned}$$

where $\doteq$ denotes an approximate equation. The linear approximation using the first- and second-order derivative at $\theta _0$ tends to approximate the cumulative score process of $\hat{\theta }$ in probability. Hjort and Koning use this Taylor expansion to derive a canonical monitoring process that approximates several independent Brownian bridges under the null hypothesis (see Hjort and Koning 2002, Eqs. (2.3) and (2.4), p. 116).

### The Approximation Error

Hjort and Koning ignore the Lagrange remainder of the Taylor expansion. The Lagrange remainder characterizes the error associated with the approximation of $\hat{\theta }$. The full Taylor expansion for a function *f* is commonly written as:

$$\begin{aligned} f(\hat{\theta }) = f(\theta _0) + f'(\theta _0) (\hat{\theta } - \theta _0) + E_2(\theta ), \end{aligned}$$

where $E_2(\theta )$ denotes the Lagrange remainder. The largest term in $E_2(\theta )$ can be described by:

$$\begin{aligned} E_2(\theta ) = \frac{f^{''}(\theta _s)}{2} (\hat{\theta } - \theta _0)^2, \end{aligned}$$

for $\theta _s$ between $\theta _0$ and $\hat{\theta }$. More specifically, the full Taylor expansion for the cumulative score process is:

$$\begin{aligned} \Psi (t\text {; } \hat{\theta }) = \frac{1}{\sqrt{n}} \sum _{i = 1}^{ \lfloor nt \rfloor } s( y_i, \theta _0) + \frac{1}{\sqrt{n}} \sum _{i = 1}^{ \lfloor nt \rfloor } i( y_i, \theta _0) (\hat{\theta } - \theta _0) + \frac{ 1}{2 \sqrt{n}} \sum _{i = 1}^{ \lfloor nt \rfloor } j( y_i, \theta _s) (\hat{\theta } - \theta _0)^2 , \end{aligned}$$

where $j( y_i, \theta )$ denotes the third-order derivative of the log-likelihood function *g*.

We will next assess the Lagrange remainder to determine the magnitude of the error associated with the linear Taylor approximation dependent on the sample size. We will discuss both facets composing the error: $(\hat{\theta } - \theta _0)^2$ and $f^{''}(\theta _s)$. First, we evaluate $(\hat{\theta } - \theta _0)^2$, where $\theta _0$ denotes the parameter estimate under . Since $\theta _0$ is unknown, we use its MLE—$\hat{\theta }$. Observe that in this case $(\hat{\theta }-\theta _0)^2$ is the standard error of the approximation and for an unbiased or asymptotically unbiased estimator it holds that $(\hat{\theta }-\theta _0)^2=\mathcal {O}_{p}(1/\sqrt{n})$. Here, $\mathcal {O}_{p}$ is the Big-O in probability notation for random variables $X_n$ and set of constants $m_n$. The notation $X_n = \mathcal {O}_{p}(m_n)$ states that there is a finite, positive *M* and an $n_0$ such that $P(|X_n/ m_n| > M) < \epsilon$ for all $n > n_0$ and any positive $\epsilon$. Thus, $(\hat{\theta }-\theta _0)^2$ is bounded by $1/ \sqrt{n}$.

Second, we evaluate $f''(\theta _s)$ which comprises the individual third-order derivatives $j( y_i, \theta _s)$ of the log-likelihood function *g*. To illustrate this derivative, we assume *g* is part of the exponential family:

$$\begin{aligned} p(x \mid \eta ) = h(x) e^{\eta ^\mathsf{T} t(x) - A(\eta )}, \end{aligned}$$

where *h*(*x*) denotes the base function, $\eta$ the natural parameter of the model, *t*(*x*) denotes the sufficient statistic, and $A(\eta )$ the log-normalizing constant—$\int _x h(x) \exp (\eta ^\intercal t(x) )\text {d}x$—that ensures that the density integrates to one. The first-, second-, and third-order derivatives of exponential family distributions w.r.t. the natural parameter are

$$\begin{aligned} \frac{\partial p(x \mid \eta )}{\partial \eta _i} = \text { }&h(x) e^{\eta ^\mathsf{T} t(x) - A(\eta )} \left( t(x)_i - \frac{\partial }{\partial \eta _i} A(\eta )\right) ,\\ \frac{\partial ^2 p(x \mid \eta )}{\partial ^2 \eta _i } = \text { }&h(x) e^{\eta ^\mathsf{T} t(x) - A(\eta )} \left( \left( t(x)_i - \frac{\partial }{\partial \eta _i} A(\eta ) \right) ^2 + \frac{\partial ^2}{\partial ^2 \eta _i} A(\eta ) \right) ,\\ \frac{\partial ^3 p(x \mid \eta )}{\partial ^3 \eta _i} = \text { }&h(x) e^{\eta ^\mathsf{T} t(x) - A(\eta )} \\&\times \left( \left( t(x)_i - \frac{\partial }{\partial \eta _i} A(\eta )\right) \left( \left( t(x)_i - \frac{\partial }{\partial \eta _i} A(\eta )\right) ^2 - 3\frac{\partial ^2}{\partial ^2 \eta _i} A(\eta ) \right) - \frac{\partial ^3}{\partial ^3 \eta _i} A(\eta ) \right) . \end{aligned}$$

The third-order derivative consists of two parts $h(x) e^{\eta ^\mathsf{T} t(x) - A(\eta )}$ and everything inside the bracket. Note that the first part is the distribution itself and is bounded to lie between zero and one. Therefore, we need to take a closer look at the second part, which mainly depends on the derivatives of $A(\eta )$. It is a convenient feature of the exponential family distributions that the moments of the sufficient statistics can be derived from the derivatives of $A(\eta )$. We will show this for the first moment, but it can be shown for all other moments.

$$\begin{aligned} \frac{\partial }{\partial \eta _i} A(\eta )&= \frac{\partial }{\partial \eta _i} \left\{ \log \int h(x) e^{\eta ^\mathsf{T} t(x) } \text {d}x \right\} \\&= \frac{\int t(x)_i h(x) e^{\eta ^\mathsf{T} t(x) } \text {d}x}{\int h(x) e^{\eta ^\mathsf{T} t(x) } \text {d}x} \\&= \int t(x)_i h(x) e^{\eta ^\mathsf{T} t(x) - A(\eta ) } \text {d}x \\&= \mathbb {E}[t(x)_i] \end{aligned}$$

Thus, if the moments of the specific exponential family distribution are bounded, the third-order derivative is bounded and $f^{''}(\hat{\theta }) = \mathcal {O}(1)$.

Taking everything together—$(\hat{\theta }-\theta _0)^2 = \mathcal {O}_{p}(1/\sqrt{n})$ and $f^{''}(\hat{\theta }) = \mathcal {O}(1)$—we obtain:

$$\begin{aligned} \Psi (t\text {; } \hat{\theta }) = \frac{1}{\sqrt{n}} \sum _{i = 1}^{ \lfloor nt \rfloor } s( y_i, \theta _0) + \frac{1}{\sqrt{n}} \sum _{i = 1}^{ \lfloor nt \rfloor } i( y_i, \theta _0) (\hat{\theta } - \theta _0) + \mathcal {O}_{p}\left( \frac{1}{ \sqrt{n}}\right) . \end{aligned}$$

This shows that the approximation error depends on the sample size, and the error will be larger for smaller samples. The approximation error tends to zero as the sample size grows. If the sample size is sufficiently large, the calculations of Hjort and Koning hold.

## Footnotes

1 The code, simulation results and online appendix can be found on the project repository https://github.com/KarolineHuth/sctpermutation.
