# Supplementary material for: A Note on the Structural Change Test in Highly Parameterized Psychometric Models
Source: Psychometrika. 2022 Feb 1;87(3):1064–80. doi: 10.1007/s11336-021-09834-6 (PMC9433362; doi:10.1007/s11336-021-09834-6)
Supplement: Supplementary file 1 — (pdf 1241 KB) [file 11336_2021_9834_MOESM1_ESM.pdf]

Online Appendix - A Note on the Structural Change Test in Highly Parameterized Psychometric Models

Huth, K.[1,2,3], Waldorp, L. J.[1], Luigjes, J.[2], Goudriaan, A. E. [2,4], van Holst, R. J. [2,3], & Marsman, M.[1]

1 Department of Psychology, University of Amsterdam
2 Department of Psychiatry, Amsterdam University Medical Center
3 Centre for Urban Mental Health, University of Amsterdam
4 Arkin Mental Health Institute

This is the online appendix for the paper "A Note on the Structural Change Test in Highly Parameterized Psychometric Models". For details on the project please refer to the original article.

Small Sample Behavior of the Structural Change Test using a Binary Auxiliary Variable

In this section we demonstrate the distribution of the p-value for the structural change test under the Null-hypothesis when testing a binary auxiliary variable. Data were simulated for a linear regression model with two, four, and eight covariates/regression coefficients k, for n is 50, 200 and 1000 cases. We sampled 5,000 datasets for each combination of k and n.

Results are shown in Figure S1. For the linear regression model, the p-value distribution is misspecified for all values of k in small samples. With increasing sample size, the p-value approximates a uniform distribution. For the Gaussian graphical model (GGM), the p-value is properly specified for models with five nodes independent of sample size. However, for larger models, the p-value distribution does not approximate a uniform distribution independent of sample size.

These findings align with the results we obtained when simulating a continuous auxiliary variable. The main difference arises for a GGM with 15 nodes. For a binary auxiliary variable, the misspecification does not improve with increasing sample size, however, for a continuous variable, the p-value misspecification noticeably improves for large samples. To conclude, the p-value distribution is misspecified independent of the type of auxiliary variable.

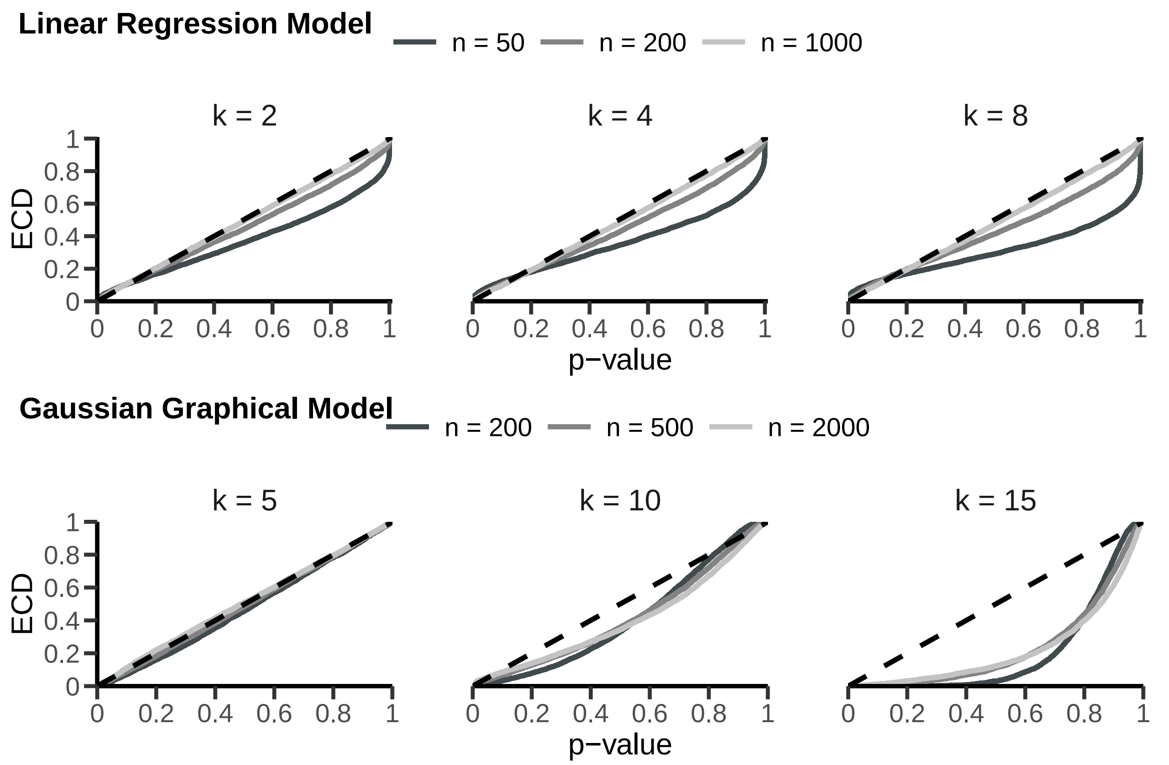

Figure S1. Empirical cumulative distributions (ECD) for the p-value under the null-hypothesis for different models and simulation settings. The auxiliary variable was simulated of binary nature. The top row shows the linear regression model results and the bottom row of the results for the GGM. Here, n represents the sample size and k the amount of covariates for the linear regression model and amount of nodes for the GGM. In each plot, the black, dashed line shows the expected uniform distribution.

Small Sample Behavior of the Structural Change Test using the Double Maximum and Crámer-von Mises statistics

In this section, we elaborate on the results of the p-value and sampling distribution for structural change tests (SCTs) when applying the double maximum (DM; see Eq. (2) in the original paper) and the Cramér-von Mises (CvM; see Eq. (3) in the original paper) test statistic. Data were simulated for a linear regression model with two, four, and eight covariates k, for n is 50, 200, and 1000 cases. We sampled 5,000 datasets for each combination of k and n.

The simulated p-value distributions are shown in Figure S2. The simulated p-value distributions in the top row are for the DM statistic, and those in the bottom row are for the CvM statistic. Observe that the p-values for the DM statistic do not follow the expected uniform distribution —shown as a black, dashed line— in the smaller sample sizes but converge to it as the sample size grows. The simulated p-value distributions for the

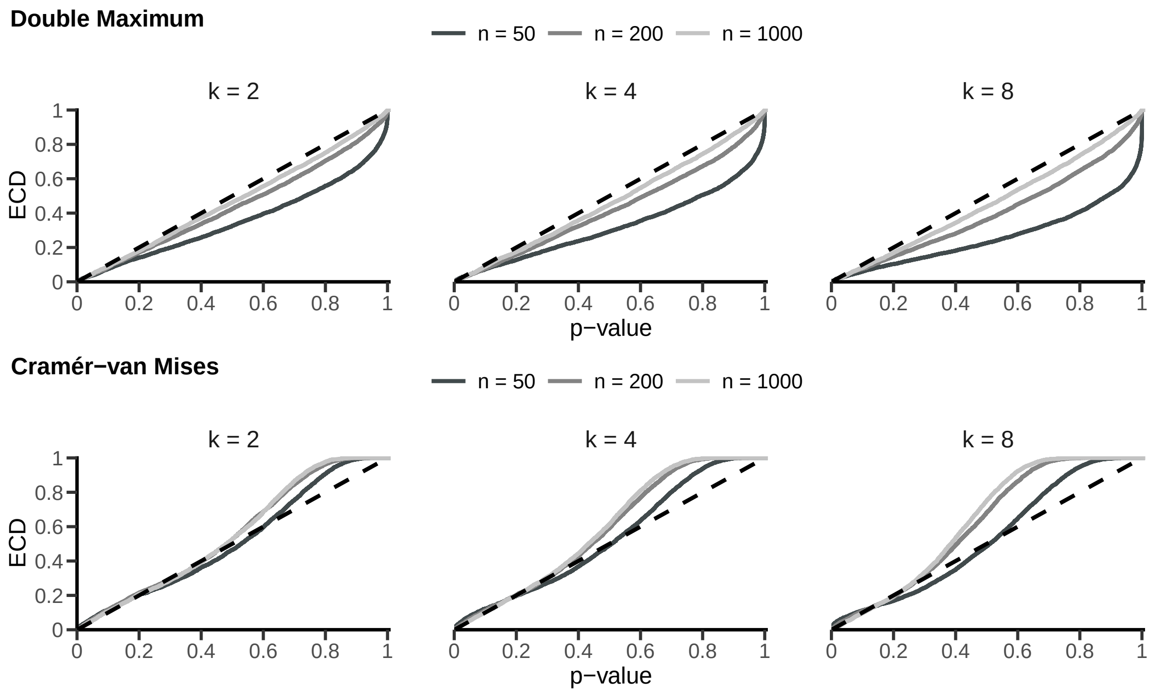

Figure S2. Empirical cumulative distributions (ECD) for the p-value under the null-hypothesis for a linear regression model for different test statistics and simulation settings. The top row shows the DM results and the bottom row the CvM results. Here, n represents the sample size and k the amount of covariates for the linear regression model. In each plot, the black, dashed line represents the expected uniform distribution.

CvM statistic also do not resemble a uniform distribution in smaller sample sizes. However, contrary to what we find for the other statistics, the p-value distribution also does not resemble a uniform distribution for larger sample sizes. The CvM p-value distributions appear to converge much slower than those of the other two statistics.

The simulated sampling distributions are shown in Figure S3 for the DM statistic and in Figure S4 for the CvM statistic. The asymptotic sampling distributions are indicated with a black solid line in these graphs. They were generated by repeatedly simulating values from a Brownian bridge and then computing the statistic on the generated data (e.g., see Andrews, 1993; Zeileis, 2006). For both statistics, the theoretical sampling distributions do not match the empirical sampling distributions for smaller sample sizes, but their fit improves for the larger sample sizes.

To conclude, the p-value and sampling distributions for the SCT appear to be misspecified for the three test statistics investigated in this paper. This means that the problems are not isolated to the use of one of the test statistics.

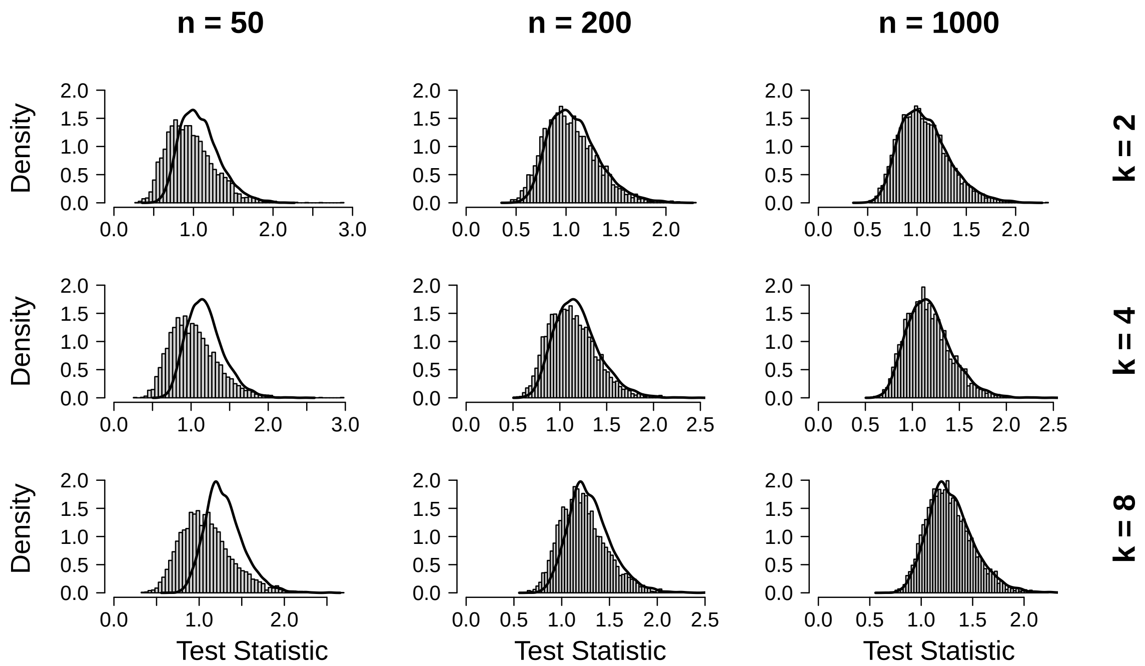

Figure S3. Distributions of the DM statistic under the null hypothesis for the linear regression model. The expected sampling distribution is depicted as a black line and was obtained by simulating observations from a Brownian bridge and applying the DM statistic to them (e.g., see Zeileis (2006)).

Assessing the Nature of the Misspecification

From the assessment thus far, it remains unclear where the misspecification comes from. The k-independent Brownian bridges approximation depends on two assumptions: the normal approximation of the scores and the accuracy of the information matrix estimate. In order to assess these two fundamental assumptions, we conducted some small scale simulations.

First, we will consider the distribution of the scores. Here, we simulated data for a GGM without any parameter invariance for two combinations of nodes (i.e., $k = 5$ and 15) and observations (i.e., $n = 200$ and $2,000$). The SCT was fitted using the R package `networktree` from which the estimated scores were extracted (Jones, Mair, Simon, & Zeileis, 2020). The distribution of scores are depicted in Figure S5 for a threshold parameter μ and in Figure S6 for an interaction parameter ρ. For the threshold parameter, the score distributions follow the expected one acceptably well, except for a GGM with 15 nodes and 200 observations. For the interaction parameters, the normal approximation of the distributions are poor; the distributions are skewed, multi-model and highly peaked. Overall, there is no difference between the distribution of the networks with fewer nodes and/or more observations, as might have been expected. Note, the distribution of the scores

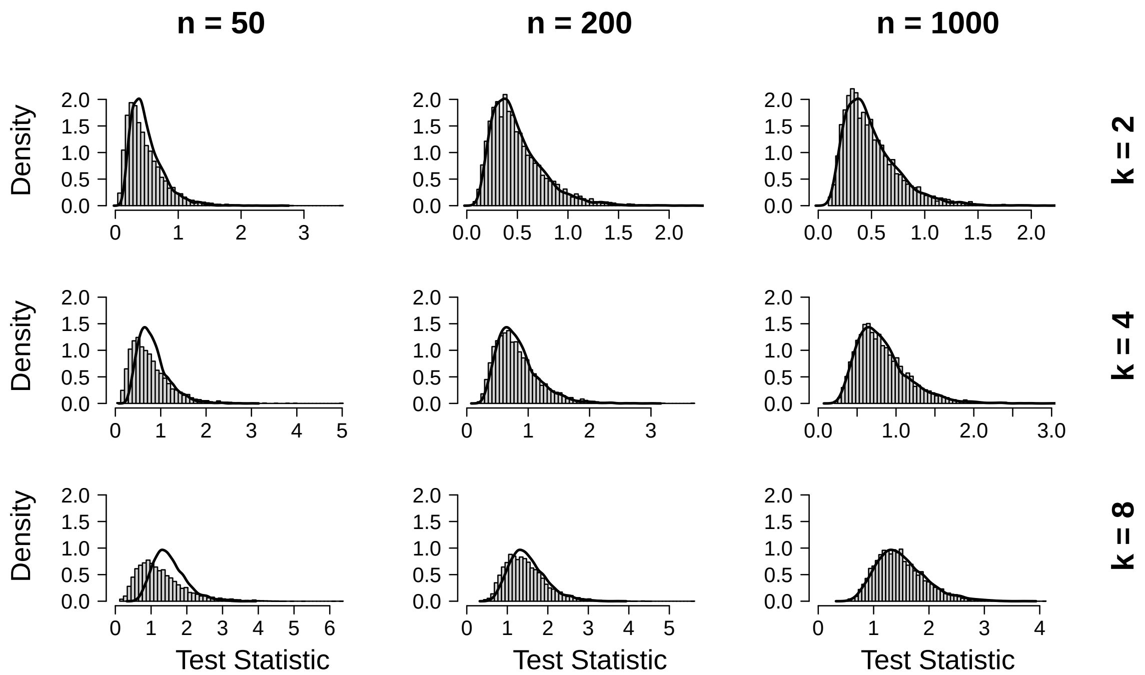

Figure S4. Distributions of the CvM statistic under the null hypothesis for the linear regression model. The expected sampling distribution is depicted as a black line and was obtained by simulating observations from a Brownian bridge and applying the CvM statistic to them (e.g., see Zeileis (2006)).

does not appear normal, however, even in situations where the distribution of the p-value under the null is correctly specified (e.g., for $k = 5$ and $n = 2,000$). Thus, the not-normally distributed scores can give an indication whether the sampling distribution and thus the p-value distribution is valid, but they do not fully explain the misspecification. Furthermore, it seems surprising that the distribution of the interaction scores is so poorly approximated, despite it being a fundamental requirement of the SCT. But this might be the central limit theorem at work.

Second, we want to assess the biasedness of the Fisher Information matrix (FIM) estimate. We simulated networks with varying number of nodes and observations and compared the true vs. the estimated FIM. We made use of the property that the FIM can be obtained through the Kronecker product of the covariance matrix (i.e., $\Sigma \otimes \Sigma$), where Σ denotes the covariance matrix and $\otimes$ the Kronecker product (Bilodeau & Brenner, 2008). The bias of the FIM was estimated as the Frobenius norm of the difference matrix of the true and estimated FIM. Results are shown in Figure S7. For a small network with 5 nodes, the bias does not depend on the sample size. In larger networks with 10 or 15 nodes the bias reduces with sample size, which is especially noticeable for the largest network (i.e., with 15 nodes).

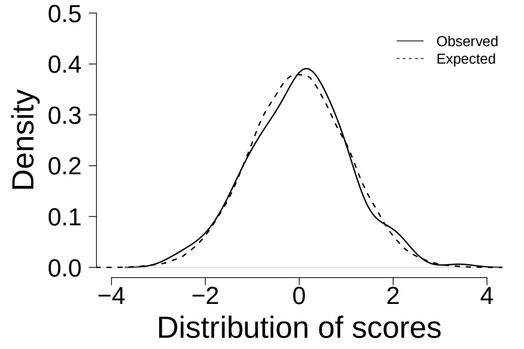

(a) Threshold parameter for $k = 5$ & $n = 200$

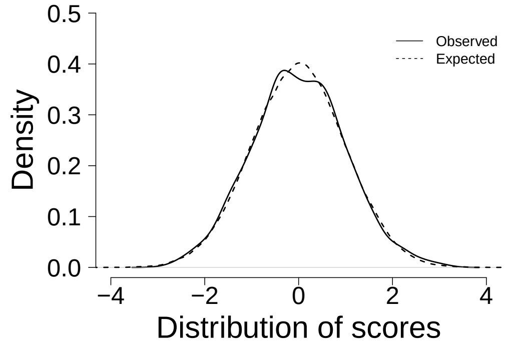

(b) Threshold parameter for $k = 5$ & $n = 2,000$

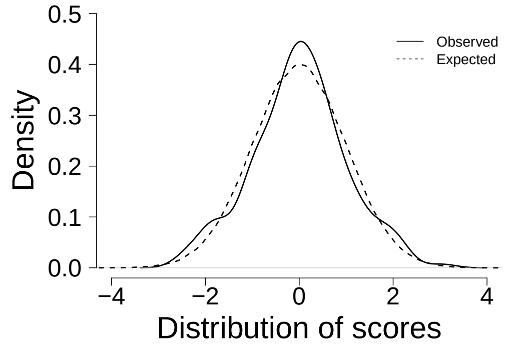

(c) Threshold parameter $k = 15$ & $n = 200$

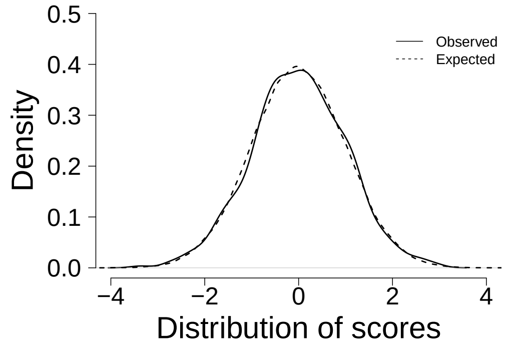

(d) Threshold parameter $k = 15$ & $n = 2,000$

Figure S5. Distribution of the scores of a threshold parameter μ, which was simulated from a GGM with various number of nodes (i.e., $k = 5$ and 15) and observations (i.e., $n = 200$ and $2,000$).

In sum, these small scale simulations gave some first insights into the nature of the issue. First, the normal approximation is poor, however, even in situations where the null-distribution is properly specified. Second, the bias of the information matrix reduces with increasing sample size for large networks. We conclude that the fundamental requirements of the test are not met and provide an avenue for more thorough investigations.

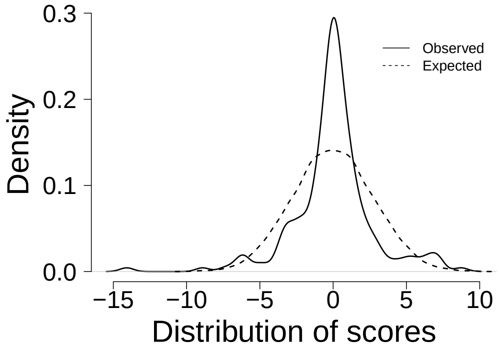

(a) Interaction parameter for k = 5 & n = 200

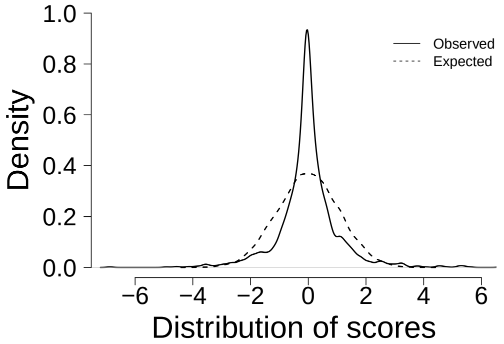

(b) Interaction parameter for k = 5 & n = 2,000

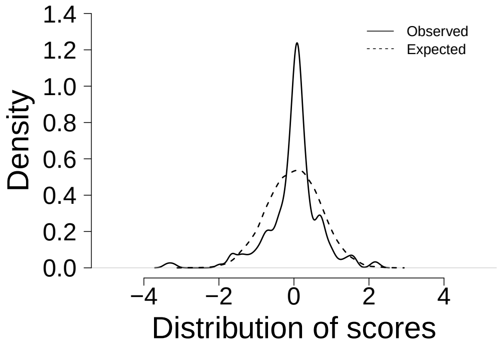

(c) Interaction parameter for k = 15 & n = 200

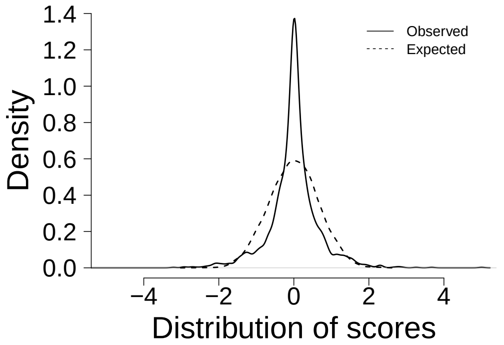

(d) Interaction parameter for k = 15 & n = 2,000

Figure S6. Distribution of the scores of an interaction parameter ρ, which was simulated from a GGM with various number of nodes (i.e., $k = 5$ and 15) and observations (i.e., $n = 200$ and $2,000$).

Small Sample Behavior of CTree under the Null Hypothesis

Contrary to the other sections, we here assess the behavior of another parameter invariance test – CTree. CTree is an alternative recursive partitioning algorithm assessing parameter invariance using permutation approaches to obtain the sampling distribution (Schlosser, Hothorn, & Zeileis, 2019). The algorithm tests whether there is any association between the transformed responses $h(Y)$ and each of the transformed splitting variables $g(Z_j)$. CTree requires the specification of an influence function $h(\cdot)$ and the transformed split variable function $g(\cdot)$. In case a parametric model is fitted to the observed data, one can obtain a model-based transformation function $h(\cdot)$, for example, a score-function. Here, CTree closely resembles the SCT, however, leveraging a conditional inference framework.

In this section we demonstrate the distribution of the p-value for CTree under the

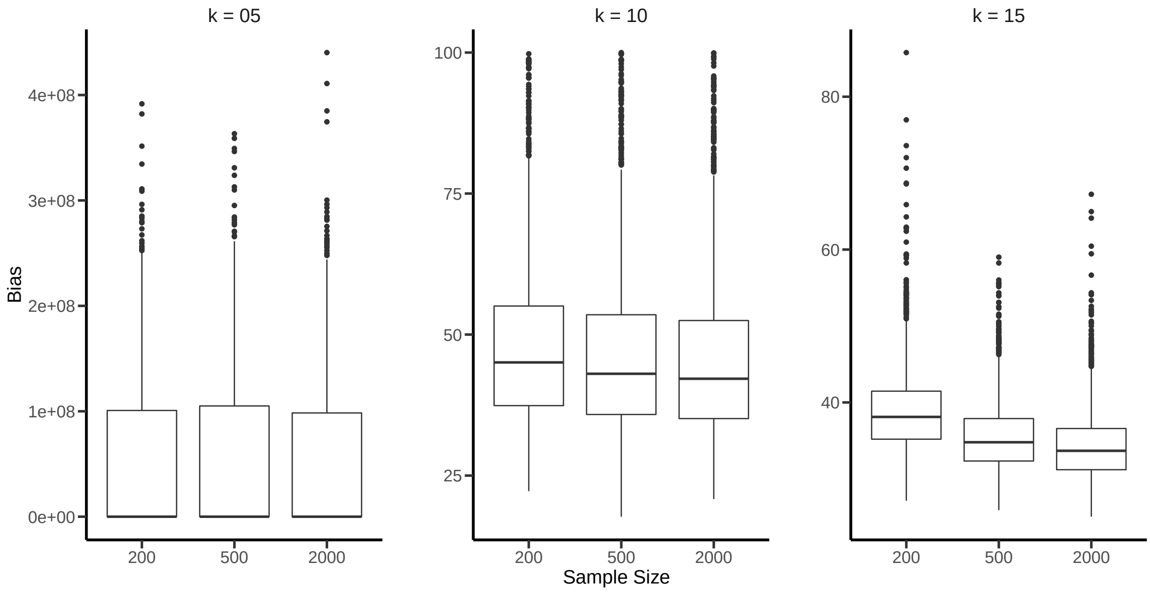

Figure S7. Bias of the estimated vs true Fisher information matrix of various sample sizes (n = 200, 500, 2000) and nodes in the network (k = 5, 10, 15).

null-hypothesis when testing a binary auxiliary variable. Data were simulated for a GGM with five, ten, and fifteen nodes k, for n is 200, 500 and 2000 cases. We sampled 1,000 datasets for each combination of k and n. Results are shown in Figure S8. The p-value distribution is uniform for all setups of k and n. CTree circumvents the small sample issues in finite samples also for large models. Thus, it can serve as an additional alternative to the permutation approach.

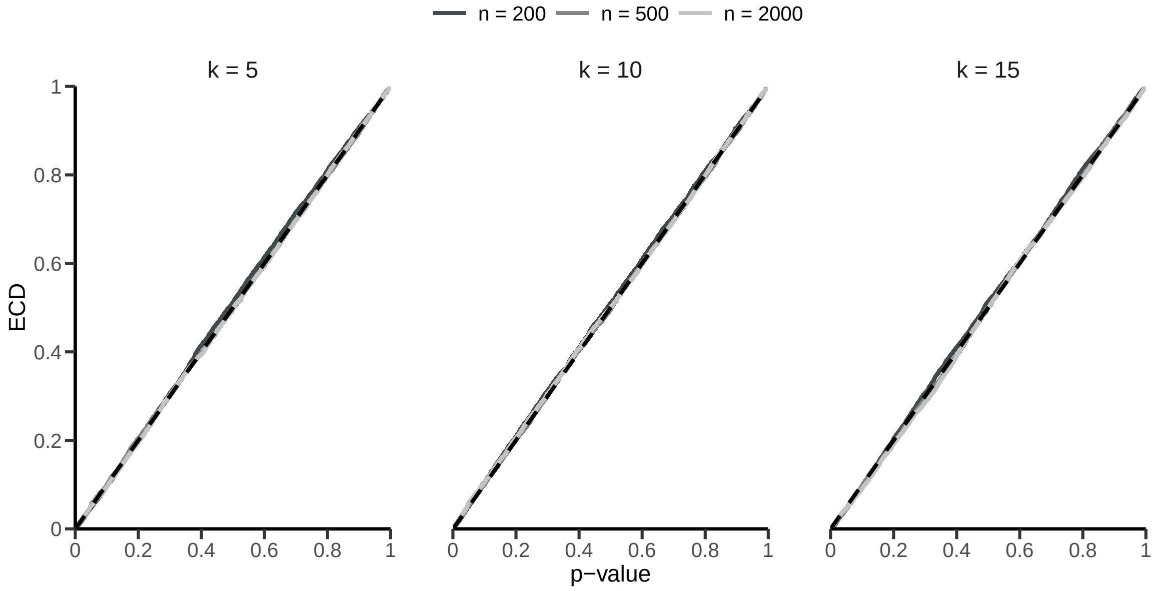

Figure S8. Distributions of the CvM statistic under the null hypothesis for the linear regression model. The expected sampling distribution is depicted as a black line and was obtained by simulating observations from a Brownian bridge and applying the CvM statistic to them (e.g., see Zeileis (2006)).

References

Andrews, D. (1993). Tests for parameter instability and structural change with unknown change point. *Econometrica*, *61*, 821–856.

Bilodeau, M., & Brenner, D. (2008). *Theory of multivariate statistics.* Springer Science & Business Media.

Jones, P. J., Mair, P., Simon, T., & Zeileis, A. (2020). Network Trees: A Method for Recursively Partitioning Covariance Structures. *Psychometrika*, *85*, 926–945. doi: 10.31219/osf.io/ha4cw

Schlosser, L., Hothorn, T., & Zeileis, A. (2019). The Power of Unbiased Recursive Partitioning: A Unifying View of CTree, MOB, and GUIDE. *arXiv:1906.10179 [stat]*.

Zeileis, A. (2006). Implementing a class of structural change tests: An econometric computing approach. *Computational Statistics & Data Analysis*, *50*, 2987–3008. doi: 10.1016/j.csda.2005.07.001
